# The role of microbiota in the pathophysiology of uterine fibroids – a systematic review

**DOI:** 10.3389/fcimb.2023.1177366

**Published:** 2023-05-26

**Authors:** Lidia Korczynska, Natalia Zeber-Lubecka, Magdalena Zgliczynska, Elzbieta Zarychta, Kornelia Zareba, Cezary Wojtyla, Michalina Dabrowska, Michal Ciebiera

**Affiliations:** ^1^ Second Department of Obstetrics and Gynecology, Centre of Postgraduate Medical Education, Warsaw, Poland; ^2^ Department of Gastroenterology, Hepatology and Clinical Oncology, Centre of Postgraduate Medical Education, Warsaw, Poland; ^3^ Department of Genetics, Maria Sklodowska-Curie National Research Institute of Oncology, Warsaw, Poland; ^4^ Department of Obstetrics, Perinatology and Neonatology, Centre of Postgraduate Medical Education, Warsaw, Poland; ^5^ Department of Obstetrics and Gynecology, College of Medicine and Health Sciences, United Arab Emirates University, Al Ain, United Arab Emirates; ^6^ International Prevention Research Institute – Collaborating Centre, Calisia University, Kalisz, Poland

**Keywords:** microbiome, microbiota, 16S rRNA, NGS, bacteria, uterine fibroid, leiomyoma, pathophysiology

## Abstract

For a long time, the uterus had been considered a sterile organ, meaning that under physiological conditions the uterus would not be colonized by bacteria. Based on available data, it may be concluded that the gut and uterine microbiome are related, and that the role of this microbiome is greater than expected. Despite being the most common pelvic neoplasms in women of reproductive age, uterine fibroids (UFs) are still poorly understood tumors whose etiology has not been fully determined. This systematic review presents the relationship between intestinal and uterine dysbiosis and uterine fibroids. A systematic review of three medical databases was carried out: the MEDLINE/PubMed, Scopus and Cochrane. In this study, 195 titles and abstracts were reviewed, including only original articles and clinical trials of uterine microbiome criteria. Finally, 16 studies were included to the analysis. In recent years, researchers dealing with reproduction in a broad sense have focused on the microbiome in various locations to study its role in the pathogenesis and, consequently, the prevention and treatment of diseases of the genital organ. Conventional microbial detection methods are not suitable for identifying bacteria, which are difficult to culture. Next-generation sequencing (NGS) provides an easier and faster and more informative analysis of bacterial populations. It seems that gut microbiota dysbiosis has the potential to be a risk factor for uterine fibroids or affect the disease process. Some changes were shown in many types of bacteria, such as *Firmicutes, Proteobacteria, Actinobacteria* and *Verrucomicrobia* detected in fecal samples in patients with uterine fibroids. In view of the few results on the link between the microbiome and uterine fibroids, further intensive studies in humans and animal models are necessary, including the possible use of different microbiome modulations in the prevention or treatment of uterine fibroids.

## Introduction

It had been believed until recently that the uterine cavity and the upper parts of the female genital organ are a sterile environment. However, subsequent studies provided strong evidence that this view was no longer valid (Chen et al., 2017; Simon, 2018). It is not surprising, especially when considering the fact that bacterial cells in the human body account for 1-3% of its total weight and are equal in number to human cells (Moreno et al., 2016).

The use of next-generation sequencing (NGS) in the analysis of hypervariable fragments of the 16S rRNA bacterial gene showed the presence of a variety of microorganisms within the uterine cavity which were usually found in the vagina and in the colon (Pereira et al., 2016; Baker et al., 2018). According to available data, the difference between the vagina, cervix, and the uterine cavity is that the colonization of the uterine cavity is relatively low (Ichiyama et al., 2021).

Fertility disorders are the driving force behind numerous studies in gynecology and reproductive medicine. For a long time, researchers have been wondering whether there are proper microbiomes for specific parts of the reproductive organ, or whether alteration in those microbiomes might have negative consequences (Brandão and Gonçalves-Henriques, 2020; Vitale et al., 2022). The effect of female genital microflora on the ability to conceive is still unclear due to the scarcity and inconsistency of published data. Nevertheless, it seems that the flora dominated by *Lactobacillus* spp. plays a key role in determining fertility, and the presence of pathogens in the genitals may disturb issues associated with it (Vitale et al., 2022). Toson et al. have recently proposed that the physiological endometrial microbiota should be considered as a group of microorganisms that allows embryo implantation and live birth, regardless of the minimal presence of pathogenic bacteria (Toson et al., 2022).

Previous studies determined the normal uterine microbiome in women of childbearing age, where *Lactobacillus* spp. played a particular role (Mitchell et al., 2015). Other types of bacteria commonly detected in endometrial/uterine swabs included *Bifidobacterium*, *Gardnerella*, *Prevotella* and *Streptococcus* (Moreno et al., 2016). Further research showed that the abundance of *Lactobacillus* might be negatively correlated with the genus *Gardnerella*, *Bifidobacterium* and *Atopobium* and positively correlated with commensal bacteria, which are very important for the immune system, e.g., *Clostridium* and *Streptomyces* (Moreno et al., 2016).

Hormonal changes affect not only the uterine muscle and the uterine mucosa itself, but they also seem to affect the microbiome found in those areas. Current data suggest that the microbiome plays an important role in female reproductive endocrine system throughout the life by interacting with estrogens, androgens, insulin, and other hormones. An imbalance in the composition of the intestinal microflora may modify the course of numerous diseases and conditions, such as pregnancy complications, polycystic ovary syndrome, endometriosis, or reproductive organ tumors (He et al., 2021; Qi et al., 2021).

Available data indicated that exogenous progesterone might significantly alter the microflora of the endometrium (Brooks et al., 2017). Interestingly, according to Brooks et al., the use of oral hormonal contraceptives may positively influence the proper functioning of the reproductive system by increasing the quantity of *Lactobacillus* spp. and reducing bacterial taxa associated with bacterial vaginosis (BV) (Brooks et al., 2017). Progesterone may increase the α-diversity of both the vaginal and endometrial microbiome. Moreover, the quantity of bacteria that may interfere with proper functioning may increase after hormonal treatment (Toson et al., 2022). Notably, naturally occurring hormonal fluctuations during the menstrual cycle correlate with the instability of the microbial population, and a regular replacement of bacterial species occurs in the vagina during the cycle (Gajer et al., 2012). Significant changes also occur in the endometrial microbiome. The increased abundance of *Prevotella* spp. and *Sneathia* spp. may constitute the features of the proliferative and secretory phases, respectively (Gajer et al., 2012).

Despite being the most common pelvic neoplasms in women of reproductive age, uterine fibroids (UFs) are still poorly understood tumors whose etiology has not been fully determined (Ciebiera et al., 2020; Yang et al., 2022). They mainly affect women of childbearing age and are diagnosed in about 70% of with European-American women and in over 80% of African women throughout their lifetime (Giuliani et al., 2020). Uterine fibroids are heterogeneous in number, composition, and size. The risk factors for those tumors seem to include age, obesity, low vitamin D levels, and endogenous and exogenous hormonal factors (Yang et al., 2022). Heavy menstrual bleeding (HMB) is the most commonly mentioned abnormal symptom associated with uterine fibroids. Other symptoms may include anemia due to increased blood loss, pressure in the pelvic cavity and on organs adjacent to the uterus, urinary and digestive complaints, as well as reproductive disorders (Stewart et al., 2016). Uterine fibroids may require surgical treatment and are a major source of gynecological and reproductive dysfunction (Navarro et al., 2021).

Given that little is known about fibroids, it is obvious that a lot is still unknown about the interaction between the endometrial and myometrial microbiome and the immune response modulating inflammatory processes within the uterus. In view of the confirmed anti-inflammatory role of *Lactobacillus* spp. in the vaginal microenvironment, it is believed that the above may also contribute to disorders of uterine homeostasis by inducing the secretion of anti-inflammatory cytokines and the production of antimicrobial peptides. Therefore, the association between uterine and endometrial dysbiosis and immune dysregulation is highly possible (Toson et al., 2022).

The intestinal flora and host’s body show a variety of reciprocal benefits. Numerous gut microbes affect the physiological functions of the host and affect the synthesis and secretion of hormones, trace elements, growth factors and immune system functions. In turn, the intestinal flora may be modified by hormonal interactions both *in vitro* and *in vivo*, thereby affecting the biological balance of the body (Rowland et al., 2018). The glucuronidase of intestinal bacteria improves estrogen reabsorption, and some intestinal bacteria can metabolize estrogen and are referred to as estrobolome (Sui et al., 2021). The high activity of estrobolome bacterial enzymes raises the level of free estrogen in the enterohepatic circulation by promoting an endogenous hormonal environment, leading to an increase in hormone levels, which can have a direct and indirect impact on the risk of developing uterine fibroids (Wang et al., 2020c). The abundance of intestinal microflora is closely correlated with the estrogenic metabolism. Numerous bacteria control estrogen content at the family and species level, with *Clostridium* and *Pneumococcus* exerting the most significant effect on estrogen metabolism (Wang et al., 2020a). Therefore, decreased estrogen levels are associated with impaired specific gut microbiota diversity (Fuhrman et al., 2014).

Pilot studies concerning the composition of the uterine cavity microbiome of women who developed fibroids showed a greater diversity of bacteria compared to control group women (Baker et al., 2018; Toson et al., 2022). It seems that fibroids may be associated with minimally altered vaginal and uterine microflora (Chen et al., 2017). For example, the endometrial microflora of patients undergoing hysterectomy for fibroids was dominated by *Acinetobacter*, *Cloacibacterium*, *Comamonadaceae* and *Pseudomonas*, while the species *Lactobacillus* were rare in the uterus (Winters et al., 2019). The results suggest that the systemic distribution of intestinal bacteria extends to the patients’ fibroids following dysbiosis or impaired intestinal barrier (Yang et al., 2022). However, the above data were obtained from few studies, and the data need to be systematized.

## Aim

The purpose of this systematic review is to provide a comprehensive summary of the fibroids and uterine microbiome literature published to date.

## Material and methods

**Figure 1:**
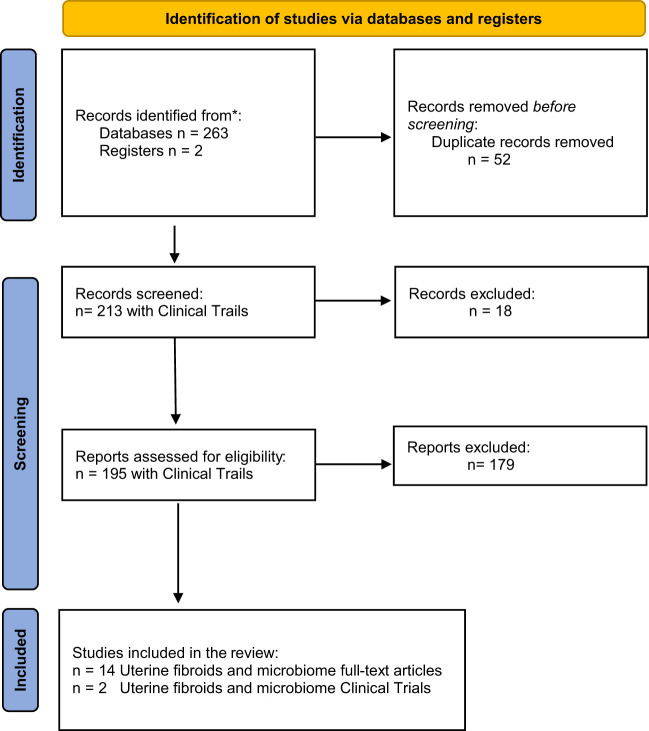
PRISMA 2020 flow diagram for new systematic reviews which included searches of databases and registers only.

A systematic review of three medical databases was carried out: the MEDLINE/PubMed, Scopus and Cochrane. The last search was conducted on January 26, 2023. The details of the search strategy are presented in **Table 1**. As a result, a total of 263 articles were obtained. Using EndNote X9 automatic duplicate search tool, 42 duplicates were identified, and then another 10 duplicates were found *via* manual search. Finally, 195 titles and abstracts were reviewed, including only original articles and clinical trials of 281 uterine microbiome criteria (**Figure 1**).

**Table 1:** Detailed search strategy in individual databases.

**Pubmed**	**64**	(“Leiomyoma”[Mesh] OR myom* OR leiomyom* OR fibromyom* OR (uterine AND fibroid*) OR (uterine AND fibrom*)) AND (“Microbiota”[Mesh] OR microbiot* OR microbiom* OR microfilm* OR flora OR microflora OR flora OR >microorganism*)
**Scopus**	187	TITLE-ABS-KEY ((myom* OR leiomyom* OR fibromyom* OR (uterine AND fibroid*) OR (uterine AND fibrom*)) AND (microbiot* OR microbiom* OR microfilm* OR flora OR microflora OR flora OR microorganism*))
**Cochrane**	12	#1 “Leiomyoma”[Mesh] 2 myom* OR leiomyom* OR fibromyom* OR (uterine AND fibroid*) OR (uterine AND fibrom*) 3 Microbiota”[Mesh] 4 microbiot* OR microbiom* OR microfilm* OR flora OR microflora OR flora OR microorganism* 5 (#1 OR #2) AND (#3 OR #4)

The asterisk (*) represents any group of characters, including no character in PubMed.

## Results

Conventional microbial detection methods are not suitable for identifying bacteria, which are difficult to culture. In turn, advanced molecular biology techniques can resolve this problem, but even such methods have limitations. The restrictions are primarily related to the possibility of detecting DNA derived from lifeless bacteria or DNA fragments. In addition, sensitive molecular biology methods, together with the low number of bacteria present in the tissue, carry a risk of contamination, which may very easily falsify the result. Experimental difficulties in the interpretation, determination of the endometrial microbiota composition are the result of conducting studies in various populations, using diverse sampling methods and a large number of research approaches as well as abundance of human DNA also create technical challenges (Abah et al., 2021). Conversely, despite numerous limitations, NGS methods seem to provide the widest variety of insights. The application of advanced sequencing and genome analysis methods resulted in rapid publications enhancing the uterine microbiota field. Today, uterine microbiota is an important research direction in human reproductive studies.

**Figure 2:**
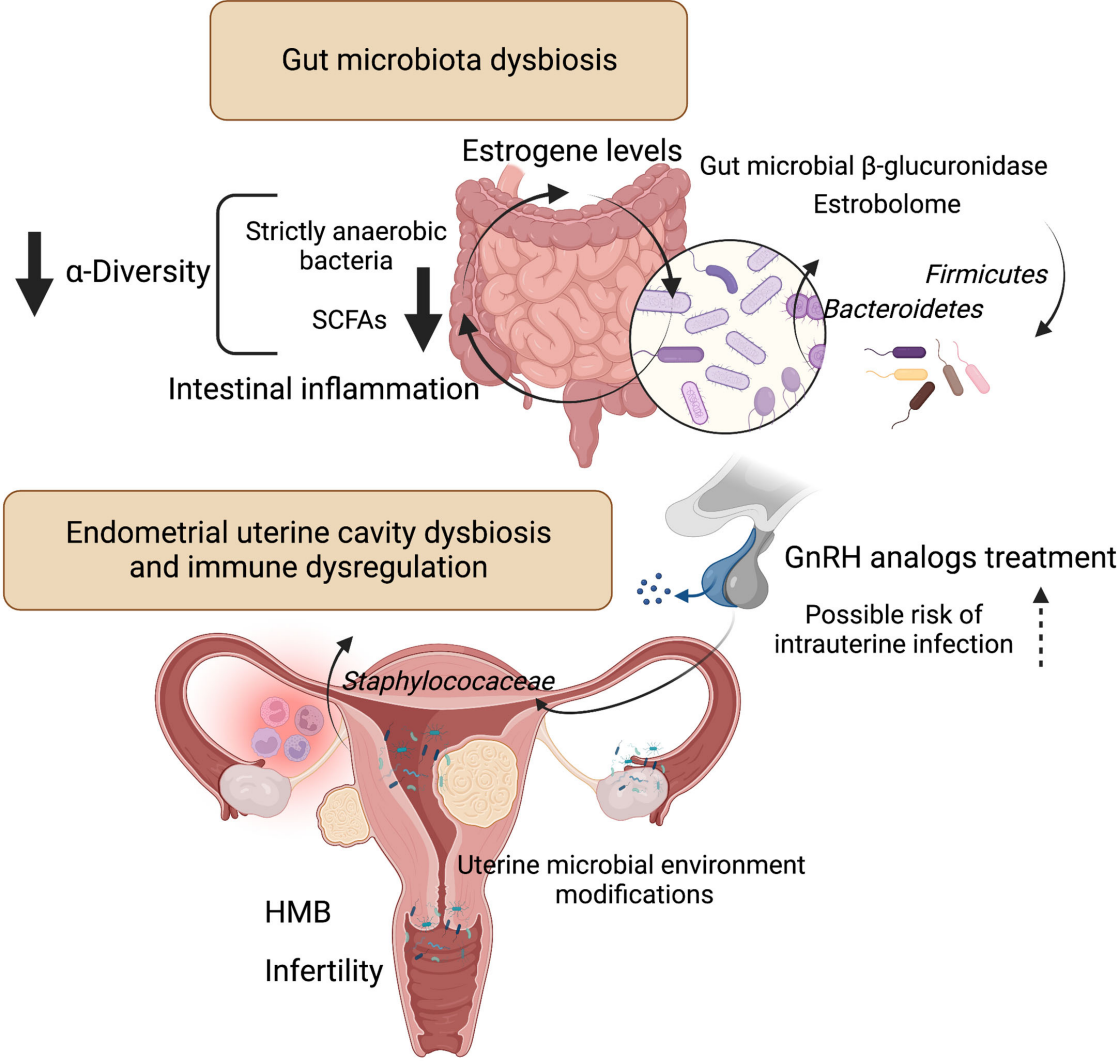
Influence of microbiota on estrogen metabolism and endometrial uterine cavity dysbiosis in patients with uterine fibroids and possible role of GnRH analogs treatment. Liver-conjugated estrogens excreted in the bile into the gastrointestinal tract could be deconjugated by intestinal bacteria that produce ß-glucosidase enzymes involved in estrogen deconjugation. This leads to their absorption into the bloodstream and allows them to bind to estrogen receptors. However, the relationship is a bidirectional - the gut microflora can also be affected by estrogen. The experimental evidence for the key role of gut bacteria in estrogen metabolism was observed decades ago. The estrobolome is defined as the gene *repertoire* of intestinal microflora with products capable to metabolize estrogens. By influencing and modulating the intestinal and hepatic circulation of estrogens, the estrobolome affects the excretion and circulation of estrogens. Numerous evidence indicates that dysbiosis of intestinal bacteria increases the chance of intestinal inflammation. This is particularly related to a decrease in strictly anaerobic bacteria and a simultaneous increase in facultative anaerobes such as Escherichia coli and Klebsiella. And SCFAs, produced mainly by strict anaerobes, have anti-inflammatory effects. According to one study (Khan et al., 2016), intrauterine higher colonization by *Staphylococaceae* was revealed in a group of control patients who used GnRH analogs. Patients with fibroids after GnRH treatment had the highest staphylococcal levels compared to other groups. **Figure 2** created with BioRender.com. Heavy menstrual bleeding (HMB); gonadotropin-releasing hormone (GnRH); short-chain fatty acids (SCFAs).

As regards uterine fibroids microbiome research, both conventional culture and NGS studies remain rather rare (**Tables 2**, **3**). Due to the limited ability to cultivate larger quantities of bacterial species in laboratory procedures, metagenomics established a popular and useful tool applied in the analysis of bacterial composition residing in the reproductive system. NGS provides an easier and faster analysis of bacterial populations, without cloning DNA sequences into a vector. Today, metagenomics represents a powerful tool used in microbiology which, *via* combining various molecular biology techniques, enables the study of the biodiversity of microbial populations and provides a better understanding of the role of individual bacteria in a particular environment, as well as the discovery of new genes (**Table 3**; **Figure 2**).

**Table 2:** Data extracted from studies using culture-based technology to investigate the uterine, vaginal, endocervical, endometrial and gut microbiome and uterine leiomyoma (uterine myoma, uterine fibroid).

Author, date, country	Study aim	Sample		Age	Detection method	Material	Study findings	Abundance of microbiota		Conclusions of the study
		Studied group, Subjects, *n*	Control group, Subjects, *n*					Increased	Reduced	
**Teisala; 1987; Finland** (Teisala, 1987)	To evaluate microbiological and histopathological findings from different levels of the endometrial cavity	10 menstruating women for hysterectomy indication 8 – uterine fibroids 2 – chronic pelvic pain		Range 36-49	Microbiological techniques	Tissue specimens from fundal, middle, and cervical area of the endometrium after removing the uterus.	Negative cultures of aerobic, anaerobic, and facultative bacteria			**Negative culture results of** ***C. trachomatis*, *N. gonorrhoeae*, *M. hominis*, *U. urealyticum.*** **Endometrial cavity of a nonpregnant uterus is sterile**.
**Bertazzoni Minelli et al.; 1990; Italy** (Minelli et al., 1990)	To assess the composition of the fecal flora in patients with breast cancer and uterine leiomyoma in comparison with a group of healthy women	18 patients with breast cancer 18 with uterine leiomyoma	30 healthy women	Range 25-52	Microbiological techniques	Stool sample, the first admission day		Anaerobic lactobacilli, streptococci (*Enterococcus faecium)* in groups of women with breast cancer and uterine leiomyoma	*Peptoniphilus asaccharolyticus* and *P. saccharolyticus* in groups of women with breast cancer and uterine leiomyoma	**Fecal bacteria reduce estrone to estradiol.** **The presence or absence of some bacterial species is important in modulating estrogen metabolism.** **The microflora may influence the metabolism of sex steroid hormones.**
**Mikamo et al.; 1993; Japan** (Mikamo et al., 1993a)	To identify the intrauterine bacterial flora in diabetic patients with various postoperative complications	Diabetic patients with abdominal hysterectomy because of uterine myoma 10 diabetic patients	Non-diabetic control patients 20 controls	Range 35-45	Quantitative bacteriological assay Anaerobic bacteria – RapID ANA II identification system (Innovative Diagnostic System, Inc., Atlanta, GA) combined with gas-liquid chromatography (GLC)	Swab from the endometrial cavity	Bacteria detected in the uterine endometrial cavity; 10 of ten diabetic patients with uterine myoma and 3 non-diabetic controls	*Enterobacteriaceae* (*Escherichia coli, Proteus* spp.*, Enterobacter cloacae*, and *Klebsiella pneumoniae*		**Antimicrobial *Enterobacteriaceae* prevention of postoperative infections in gynecologic procedures in diabetic patients.**
**Mikamo et al.; 1993; Japan** (Mikamo et al., 1993b)	To identify the intrauterine bacterial flora in patients with uterine endometrial cancer	Patients with uterine endometrial cancer 20 – uterine endometrial cancer	Patients without complications other than uterine myoma 20 controls	Range 44-69	Quantitative bacteriological assay Anaerobic bacteria – RapID ANA II identification system (Innovative Diagnostic System, Inc., Atlanta, GA) combined with gas-liquid chromatography (GLC)	Endometrial cavity with a polyester fiber swab	*Enterobacteriaceae*, *Streptococcus agalactiae* and anaerobic bacteria detected in all patients with uterine endometrial cancer Patients without complications other than uterine myoma – no detection of bacteria	*Enterobacteriaceae, Streptococcus agalactiae* and anaerobic bacteria		Products ofaerobicand anaerobic bacteria considered to contribute to endometrial carcinogenesis. Uterine endometrial cancer provides favorable conditions for bacterial growth.
**Møller et al.;1995; Denmark **(Møller et al., 1995)	To evaluate whether the uterine cavity is non-sterile, in contradiction to previous suggestions	99 women admitted for hysterectomy 34 patients with uterine fibromyoma 29 patients with persistent irregular vaginal bleeding 10 patients with malignancy of the cervix (carcinoma *in situ* cervicis uteri)		Range 29-84	Microbiological techniques Histological examination	Cervical specimens Endometrial specimens	25% of all the patients harbored one or more microorganisms in the uterus	*Gardnerella vaginalis*, *Enterobacter* and *Streptococcus agalactiae*		Uterine cavity colonized with potentially pathogenic organisms. Inflammation of the uterine cavity should be evaluated *via* hysteroscopic examination before hysterectomy.

**Table 3:** Data extracted from studies using sequencing-based technology to investigate the uterine, vaginal, endocervical, endometrial and gut microbiome and uterine leiomyoma (uterine fibroid).

Author, date, country	Study aim	Sample		Age	Detection method	Material	Study findings	Abundance of microbiota		Conclusions of the study
		Studied group, Subjects, *n*	Control group, Subjects, *n*					Increased	Reduced	
**Khan et al.; 2016; Japan** (Khan et al., 2016)	To investigate microbial colonization in the intrauterine environment and cystic fluid of women with and without endometriosis	32 women with endometriosis	32 patients with uterine myoma	Range 21-52	16S rDNA sequence Illumina Miseq	Endometrial swabs Cystic fluid	Multiple bacteria detected in the endometrial swabs and cystic fluid collected from women with and without endometriosis	*Lactobacillaceae* in patients with uterine myoma	*Streptococcaceae, Staphylococcaceae, Enterobacteriaceae* in patients with uterine myoma	Sub-clinical infection in the intrauterine environment and in the cystic fluid of ovarian endometrioma.
**Walther-Antoínio** **et al.; 2016; USA** (Walther-António et al., 2016)	To investigate the uterine microbiome and its putative role in endometrial cancer	17 patients with endometrial cancer 4 patients with endometrial hyperplasia 10 patients with benign uterine conditions		18 years of age or older	16S rDNA V3-V5 region Illumina MiSeq	Vaginal swab, cervical swab, biopsies: fallopian, ovarian, peritoneal Urine samples	Structural microbiome shift in the cancer and hyperplasia cases, distinguishable from the benign cases	*Firmicutes (Anaerostipes, ph2, Dialister, Peptoniphilus, Ruminococcus*, and *Anaerotruncus), Spirochaetes (Treponema), Actinobacteria (Atopobium), Bacteroidetes (Bacteroides* and *Porphyromonas)*, and *Proteobacteria (Arthrospira)*		Suspected dominnation of *Stenotrophomonas* in UFs
**Chen et al.; 2017; China** (Chen et al., 2017)	To investigate potential bacterial markers for adenomyosis and endometriosis	110 women of reproductive age		Range 22-48	V5 to V4 Region Ion Torrent Personal Genome Machine Real-time qPCR Conventional bacterial culturing	Nylon flocked swabs from 6 locations (the vagina, cervical mucus, cervical canal, endometrium, fallopian tubes, fluid from the pouch of Douglas)	Over- represented microbial taxa, correlated with potential functions of the menstrual cycle in patients with adenomyosis or infertility due to endometriosis	Vaginal and cervical samples: *L. iners* in patients with hysteromyoma	Vaginal and cervical samples: *Lactobacillus* sp. in patients with hysteromyoma	Vaginal or cervical microbiota useful in the detection of common diseases in the upper reproductive tract.
**Wang et al.; 2020; China** (Wang et al., 2020b)	To investigate the effect of transabdominal hysterectomy on the diversity of the intestinal flora in patients with uterine fibroids	15 preoperative patients with uterine fibroids	15 postoperative patients with uterine fibroids	Range 40–45	High-throughput sequencing of the 16S rRNA gene Illumina HiSeq	Stool	Decreased abundance and diversity of the intestinal flora after abdominal hysterectomy	*Proteobacteria* after abdominal hysterectomy		After abdominal hysterectomy: reduced diversity and abundance of the intestinal flora; lower level of estrogen in the body after abdominal hysterectomy.
**Riganelli et al.; 2020; Italy** (Riganelli et al., 2020)	To explore structural variations of vaginal and endometrial microbiota in embryo implantation failure	34 patients undergoing personalized hormonal stimulation 1 – with G1 uterine fibroid		Range 22–43	16s rRNA V4-V5 Illumina MiSeq	Vaginal fluid, and endometrial biopsy	Significant difference between vaginal and endometrial microbiota	*Lactobacillus* in pregnant women		Uterine microbiota structurally differed from the vaginal microbiota. Reduction of barriers resulting from translocation from the vagina to the endometrium. Predictive “microbiota dysbiosis” before assisted reproductive technology (ART) treatment.
**Liu et al.; 2021; China** (Liu et al., 2021)	To evaluate how gut microbiota affects host immune response and induces an imbalance in cytokine levels	41 miscarriage patients 4 – with uterine fibroids	19 controls	Mean age 31.3 ± 5.0	The V3-V4 variable regions of the 16S rRNA gene llumina MiSeq PE300 Fecal metabolic profiling using liquid chromatography/mass spectrometry (LC/MS) Cytokine quantification by flow cytometry	Stool	Microbial diversity reduced in the miscarriage patient group Microbe-associated metabolites (imidazolepropionic acid) Positively associated with changes in the levels of Th1/Th17 cytokines in the miscarriage group		*Prevotella_1, Prevotellaceae_UCG_003* and *Selenomonas* in the miscarriage group	The role of gut microbiota, stool metabolites and Th1/Th17-mediated immune response in miscarriage patients.
**Kim et al.; South Kora; 2022** (Kim et al., 2022)	To evaluate the clinical relationship between the vaginal microbiome and the pathophysiology of recurrent vaginitis (RV)	40 patients of reproductive age with RV	100 healthy women	Range 20- 55	16S ribosomal RNA gene	Flocked swab	Bacterial abundance significantly lower in patients with RV Species evenness and diversity significantly higher in patients with RV Beta diversity significantly different between patients with RV and healthy individuals Higher species richness and diversity in patients with underlying uterine diseases (uterine leiomyoma, adenomyosis, and endometrial polyps)		*Lactobacillus* spp. patients with RV	Changes in vaginal microbial community strongly associated with RV. Vaginal microbiome is valuable for detecting and treating gynecological diseases in the future.
**Hua et al.; 2022; China** (Hua et al., 2022)	To analyze alterations of the cervical canal microbiota in intrauterine adhesion (IUA) patients	23 patients with mild-to-severe IUA 8 women with infertility 3 women with submucous myomas 8 women with endometrial polyps		18 years of age or older	16S rDNA high-throughput sequencing	Cervical mucus	Lower diversity of bacteria in the group with moderate or severe IUA	*Firmicutes* in IUA patients *Firmicutes*/*Acinetobacteria* or genus *Lactobacillus/Gardnerella* in the severity of IUA		The severity of IUA associated with a higher bacterial load but lower diversity.
**Mao et al.; 2022; China** (Mao et al., 2022)	To investigate possible differences in gut microbiome compositions between patients with uterine fibroids (UFs) and healthy control subjects	42 patients with uterine fibroids	43 control subjects	Range: patients 24–52 controls 23–54	16S rRNA quantitative arrays	Stool	Significantly lower diversity in patients with UFs The microbial composition of UF patients deviated from that in healthy controls.	*Pseudomonas stutzeri*, *Prevotella amnii* in patients with UFs	*Bifidobacteria scardovii, Ligilactobacillus saerimneri*, *Lactococcus raffinolactis* in patients with UFs	Gut microbiota dysbiosis has the potential as a risk factor. UFs associated with alterations of gut microbiome diversity. Host–gut microbiota – role in the development and prevention in UF pathogenesis.

Search Results on ClinicalTrials.gov website revealed 426 studies for: Myoma/Fibroid; Uterus terms (**Table 4**). The issue of the uterine microbiome was tackled in 7 of them, and only 2 without the exclusion criteria of fibroid, leiomyoma or myoma (**Table 5**).

**Table 4:** Myoma/Fibroid; Uterus terms of 426 studies (*ClinicalTrials.gov* website, February 2023).

Terms	Search Results (studies)*	Entire Database (studies)**
Synonyms		
**Uterine Fibroid**	426	426
**Leiomyoma**	407	407
**myofibroma**	320	320
**Fibroid**	313	313
**Uterine myoma**	42	42
**uterus myoma**	3	3
**Fibromyoma**	3	3
**Uterine Fibromas**	2	2
**Fibroid**	426	426
**Leiomyoma**	407	407
**myofibroma**	320	320
**Uterine myoma**	42	42
**uterus myoma**	3	3
**Fibromyoma**	3	3
**Uterine Fibromas**	2	2

–No studies found; *Number of studies in the search results containing the term or a synonym; **Number of studies in the entire database containing the term or a synonym.

**Table 5:** Clinical trials of the uterine microbiome, without the exclusion criteria of fibroid, leiomyoma, myoma (*ClinicalTrials.gov* website, February 2023).

Identifier	Sponsor	Condition	Intervention/treatment	Study Type	Estimated enrollment	Aim	Material	Criteria		Outcome Measures
								Inclusion	Exclusion	
**NCT05337072**	Universitair Ziekenhuis Brussel	Endometritis; Chronic subfertility	Diagnostic Test: Microbiome study	Observational	1000 participants	To gain insight into the microorganisms that are present in the female reproductive tract based on various techniques	Endometrial biopsy and a vaginal swab	•Women planned for IVF/ICSI treatment •Women who undergo a diagnostic hysteroscopy in preparation for their treatment	•interventional hysteroscopy planned •previous history of chronic endometritis •use of antibiotics in the last 3 months	•Microbiome evaluated through metagenomics sequencing and high-throughput culturomics
**NCT03405883**	University Hospital, Ghent	Repeated implantation failure; Normal fertile	Vaginal swab and endometrial biopsy	Observational	40 participants	To characterize the uterine microbiome in women with repeated implantation failure as well as in normal fertile women	Obtained vaginal swab and endometrial biopsy in the midluteal phase of the cycle	•18-40 years old (max 39 years and 364 days at the day of signing the informed consent) •Negative serological tests for HIV, HBV, HCV, RPR for syphilis	•Hormonal contraception •Intra-uterine device use •Antibiotic treatment in the current cycle	Midluteal vaginal and endometrial microbiome profile Microbiome analysis using 16S ribosomal RNA sequencing

## Discussion

The presence of the microbiota in the body is known to be important for human health. In recent years, advances in molecular biology techniques have not only allowed the confirmation of the occurrence of microorganisms in the human digestive, respiratory and urinary systems, but also prompted the detection of the presence of microorganisms in organs previously considered sterile, such as the uterine cavity (Dekaboruah et al., 2020).

For a long time, the uterus had been considered a sterile organ, meaning that under physiological conditions the uterus would not be colonized by bacteria. In 1950s, research based on bacterial culture methods revealed the first reports suggesting that the uterus was an organ with its own microbiota. Further development of diagnostic techniques and molecular biology methods in early 21st century allowed the reanalysis of the composition of the uterine microbiota, including bacteria which are not easily cultured under laboratory conditions (Franasiak et al., 2021).

Until the late 1980s, the results of most studies related to the microbiome of the myomal uterine cavity had indicated the sterility of the uterus (Teisala, 1987; Mikamo et al., 1993b) (**Table 2**). Several years later, a quantitative bacteriologic assay of swabs from the endometrial cavity after myoma uteri hysterectomy demonstrated an increased amount of *Enterobacteriaceae* family, including *Escherichia coli, Proteus* spp.*, Enterobacter cloacae, and Klebsiella pneumoniae* (Mikamo et al., 1993a). In contrast, the authors expressed a different view of the microbiome of the uterine cavity affected by cancer. The results showed the distribution of *Enterobacteriaceae, Streptococcus agalactiae* and anaerobic bacteria detected in all patients with uterine endometrial cancer (Mikamo et al., 1993b). In conclusion, the products of aerobic and anaerobic bacteria contributing to endometrial carcinogenesis as well as uterine endometrial cancer provided preferential environment for bacterial growth. A turning point occurred when a study of nearly 100 women was conducted and revealed that myomal uterine cavity was colonized with potentially pathogenic organisms (Møller et al., 1995). In samples from the uterine cavity, a quarter of the women cultured one or more bacteria using microbiological techniques. *Gardnerella vaginalis, Enterobacter* and *Streptococcus agalactiae* pathogenic bacteria were identified. The deviations of obtained results were due to non-cultivation of bacteria under laboratory conditions or the presence of only lifeless bacteria in the material.

The modulating effect of the gut microbiome on the uterine cavity was highlighted in the 1990s. Using culture techniques, the composition of the stool microbiome was examined in patients with breast cancer and uterine leiomyoma (Minelli et al., 1990). The study showed fecal bacteria reduction of estrone to estradiol (Minelli et al., 1990). The findings confirmed the decreased amount of *Peptoniphilus asaccharolyticus* and *P. saccharolyticus* and an increased number of anaerobic lactobacilli and *Enterococcus faecium* in the gut microbiome of breast cancer and uterine leiomyoma women. The presence or absence of some bacterial species is important in modulating estrogen metabolism. Thus, the microbiome influenced the metabolism of sex steroid hormones (Hussain et al., 2021).

It has been known for a long time that the genus *Lactobacillus* represents the dominant component of the vaginal microbiota and plays a protective role for the vaginal microenvironment. The presence of Lactobacilli was also confirmed in the upper female reproductive tract (FRT) (Verstraelen et al., 2016; Chen et al., 2017; Benner et al., 2018). Chen et al. demonstrated the predominance of *Lactobacillus* bacteria (> 97.56%) in the cervix. In the uterus, *Lactobacillus* (30.6%), *Pseudomonas* (9.09%), *Acinetobacter* (9.07%), *Vagococcus* (7.29%), and *Sphingobium* (5.0%) were identified, while the fallopian tubes revealed the presence of *Acinetobacter* (18.27%), *Comamonas* (11.49%), *Pseudomonas* (9.9%), *Pseudomonadaceae* (9.1%), and *Dysgonomonas* (5.11%) (Chen et al., 2017). Further studies published in 2018 and 2019 confirmed the occurrence of bacteria from the *Lactobacillaceae* family not only in the vagina, but also in other segments of the female reproductive tract (Moreno et al., 2016; Koedooder et al., 2019). The protective role of *Lactobacillus* in the vagina was recognized. However, the precise mechanism of the effect and a possible impact on fertility have not been fully elucidated and require further research.

Today, we know that a gigantic number of human microbes influences the physiological functions of the human body at all times and has a profound effect on the synthesis and secretion of hormones and various growth factors. As a result, changes in the composition of the human gut flora may affect the body in various aspects, including the immune or hormonal system (Thursby and Juge, 2017). It is well known how important hormones (estrogens and progesterone) are in the pathophysiology of fibroids (Bulun, 2013). The associated growth factors are equally important, with transforming beta growth factor probably being the crucial one (Ciebiera et al., 2017).

Leaving aside the above words of introduction concerning new generation research and general disorders related to female reproductive tract colonization, our analyses of publications mostly indicate that almost no data are available on the microbiota and uterine fibroids. It seems almost unlikely assuming that uterine fibroids are the most common problem with which women present to gynecologists (Stewart et al., 2016). This problem has a large clinical impact because most papers that may be reviewed are not analyzed in terms of fibroids. The analyses concentrated on the fact that fibroids were not an exclusion criterion for other studies. Therefore, our conclusions will certainly not be strong.

We identified 4 studies that have attracted our greatest attention in the analysis and that seem to be more significant in terms of the pathophysiology of uterine fibroids and possible clinical situations associated with them. We would like to focus on those publications below.

The first study on the uterine cavity microbiota was published in 2016 by Khan et al. The Japanese authors conducted a molecular analysis of intrauterine colonization with microorganisms of women with endometriosis (Khan et al., 2016). The study included a total of 64 women half of whom had endometriosis and the other half was not affected by the condition. In each group, 16 patients additionally received GnRH analogs for 4-6 months. The results were highly interesting. A high percentage of bacteria was detected in the swabs collected from the endometrium and from the fluid obtained from cysts. Metagenomic tests showed that the proportion of *Lactobacillaceae* was significantly reduced, while the proportion of *Streptococcaceae, Staphylococaceae, and Enterobacteriaceae* was significantly increased in the GnRH analog-treated groups of women with endometriosis compared to women who did not receive those drugs. Ordinary tests did not allow for the assessment of cystic fluid for microbial colonization. The results of the 16S metagenomic test were different. A much higher percentage of *Streptococcaceae* (p<0.01) and *Staphylococaceae* (p<0.05) was detected in the fluid collected from cysts of women with ovarian endometriosis compared to fluid collected from cysts without an endometriotic etiology. It is difficult to determine what effect those data have on uterine fibroids. However, a significantly higher colonization by *Staphylococaceae* (p<0.05) and an insignificant statistical difference for colonization by *Enterobacteriaceae* was revealed in a group of control patients who used GnRH analogs (due to uterine fibroids) compared to samples obtained from control group women who were not pharmacologically treated. Interestingly, according to the data obtained from the study, the group of patients with fibroids after GnRH treatment had the lowest overall percentage of streptococcal colonization and the highest Staphylococcal levels compared to other groups. It is not known what these data mean from the viewpoint of uterine fibroids and what the clinical application may be. However, the role of drugs blocking the pituitary was shown not only to consist in regulating the growth of fibroids, but also to change the entire uterine environment, which may affect other important features such as fertility or a possible relationship with pregnancy. It certainly requires a lot of well-designed research, in which not endometriosis, but uterine fibroids will constitute the focus (Linder et al., 2021; Maxim et al., 2022). The authors concluded that the results indicated the presence of a subclinical infection in the intrauterine environment and, importantly, in the fluid collected from the lesions in the ovary. Furthermore, the authors emphasized the side effect of treatment with GnRH analogs associated with promoting an intrauterine and/or ovarian infection (Khan et al., 2016)

The second of the studies analyzed in the context of uterine fibroids concerned the effect of transabdominal hysterectomy on the intestinal flora in patients with uterine fibroids using high-throughput sequencing (Wang et al., 2020b). According to the authors, as could be predicted, estrogen levels decreased after transabdominal hysterectomy. However, post-hysterectomy high-throughput sequencing showed that the quantity and diversity of intestinal flora decreased.

It is known that total hysterectomy is one of more commonly proposed procedures in women with uterine fibroids who do not have fertility requirements. A group of doctors obviously struggle with such an approach and try to convince their colleagues to switch to more conservative surgical methods, use non-operative methods such as thermoablation (Lozinski et al., 2021) or switch to pharmacological treatment (Niaz et al., 2022). Complete hysterectomy affects the level of sex hormones. According to available data, serum AMH levels might be decreased after hysterectomy, with a greater reduction when total hysterectomy is performed in comparison with supracervical hysterectomy (Yuan et al., 2015). It is due to the fact that the anatomical structure of the uterus and ovary is closely correlated. A significant portion of the blood supplied to the ovary comes from the ascending branch of the uterine artery. Simultaneously, regardless of anatomical changes, thermal damage is common during procedures, which can impair the blood supply to the ovaries after complete hysterectomy, thus leading to a reduction in the secretion of sex hormones (Wang et al., 2020b).

So what is the significance of the results of the study by Wang et al.? Previous research demonstrated that a decrease in estrogen levels leads to a decrease in the diversity of the intestinal flora and a reduction in the abundance of thick-walled bacteria, such as *Clostridium* (Breban, 2016). Decreased estrogen levels in the study described leads to other situations, because of an increase in the quantity of *Firmicutes*, a decrease in the diversity of *Bacteroidetes* and an increase in the species diversity of *Proteobacteria*. The authors concluded that transabdominal hysterectomy could reduce the level of estrogen in the body and reduce the diversity and abundance of intestinal bacterial flora before and after surgery with the growth of *Proteobacteria* being the main difference in that case (Wang et al., 2020b). Obviously, it probably cannot be said that the mechanism underlying this regulation has already been determined. Only a deeper analysis can help understand whether the intestinal microflora may affect the risk of uterine fibroids by affecting endogenous estrogens (Yang et al., 2022).

Another analyzed study tackled the issue of interactions between the intestinal microflora and cytokine disorders in women with unexplained causes of miscarriages. Five patients with fibroids were included in this study, according to the data provided. Regrettably, this group did not undergo any additional individual analyses (Liu et al., 2021). Multiple authors suggested that dysregulation within the cytokine and growth factor network is involved in the pathogenesis of unexplained pregnancy loss (Calleja-Agius and Brincat, 2008). As already known from the previous paragraphs, the microbiota affects the host’s immune response. However, the question of how this dysbiosis impairs he cellular immune function in the event of miscarriage remains ambiguous. It is also known that factors related to the Th1 response, such as tumor necrosis factor alpha (TNF-α), increase in the serum of patients affected by miscarriage (Mallmann et al., 1991). TNF-α was also reported to inhibit trophoblast invasion and elevated TNF-α levels were identified in women who experienced miscarriage (Azizieh and Raghupathy, 2015). According to a large amount of data, TNF-α also seems to be an extremely important cytokine in the biology of uterine fibroids, associated symptoms and conditions (Ciebiera et al., 2018b). It was shown that its concentration was increased in women with symptomatic uterine fibroids (Ciebiera et al., 2018a; Kali and Cagiran, 2022). Currently available data suggest the occurrence of an inflammation-like condition in women with uterine fibroids, with TNF-α being a strong inducer of the state (Ciebiera et al., 2018b).

Liu et al. (Liu et al., 2021) demonstrated that the microbiological diversity and a relative abundance of *Prevotella*_*1*, *Prevotellaceae_UCG_003* and *Selenomonas_1* were significantly reduced in cases of miscarriage. Further analyses indicated that some microbial-related metabolites were positively associated with changes in Th1/Th17 cytokine levels in the miscarriage group. The authors concluded that a network between the intestinal microbiota, fecal metabolites and Th1/Th17 might mediate miscarriage recurrences. Moreover, the intestinal microflora in patients with a history of miscarriage showed a much higher concentration compared to controls. Given that only a few patients with uterine fibroids participated in this study, it is not possible to draw unambiguous conclusions about this particular condition. However, a number of factors and pathways, e.g., hormonal-immune ones, fit quite well into the same ones that affect uterine fibroids. Seemingly, it may be a very promising topic of research, e.g., to study why some fibroids may increase the risk of miscarriages or improper implantation.

Finally, the last study was designed for a group of patients with uterine fibroids. The authors used 16 rRNA high-throughput microarrays to compare gut microbiological differences between healthy women and women with uterine fibroids. Moreover, the authors aimed to determine the correlations and interactions between the intestines, microbiome and uterine fibroids (Mao et al., 2022). The study included 42 patients with uterine fibroids and 43 patients without a history of fibroids. The authors demonstrated very interesting correlations. Firstly, microbiome diversity in patients with uterine fibroids was significantly lower than in healthy controls. The microbiological composition of samples collected from patients with uterine fibroids differed from that of healthy women. Some changes were shown in many types of bacteria, such as *Firmicutes, Proteobacteria*, *Actinobacteria* and *Verrucomicrobia* detected in fecal samples in patients with uterine fibroids.

Further analysis of bacterial abundance showed that some species were present to a smaller extent (e.g., *Bifidobacteria scardovii, Ligilactobacillus saerimneri* and *Lactococcus raffinolactis*) and some were more abundant (e.g., *Pseudomonas stutzeri* and *Prevotella amnii*). Moreover, microbial interactions and specific networks in uterine fibroids exhibited lower connectivity and complexity as well as higher clustering properties compared to the second group. The results indicated it was highly possible that intestinal dysbiosis might be a significant risk factor for the development or growth of uterine fibroids. The data confirmed that uterine fibroids might be strongly associated with changes in the diversity of the gut microbiome. This provides a new direction for further analyses of the intestinal microflora and its links to the development and growth of uterine fibroids. The results will certainly provide an incentive to develop new research and further analyses. Furthermore, as the authors noted, they may also constitute pioneering reports in terms of new therapies for uterine fibroids, such as the development or use of probiotics to protect against uterine fibroids or support therapy (Mao et al., 2022).

Apart from the scarcity of publications on the microbiota and uterine fibroids, it is also quite surprising that there are practically no registered studies that could expand such knowledge. In preparing this systematic review, the authors also reviewed the clinicaltrials.gov database and, to their surprise, it turned out that only 7 out of 426 studies related to the microbiome were related to the uterine microbiome (only 2 were directly linked, and only 2 indirectly addressed the issue of uterine fibroids). As regards those two clinical studies, the first one concerns chronic endometritis and its effects on fertility and pregnancy. This study is particularly important because chronic endometritis is commonly diagnosed in the context of fertility problems, and the patient is often treated blindly with broad-spectrum antibiotics in this case. Uterine fibroids are not simply an exclusion criterion here and such patients can be recruited for the study (**Table 5**). The topic of the second study is different, i.e., it addresses implantation failures. The study focuses on the characteristics of the uterine microbiome in women with repeated implantation failures and in women with normal fertility. The absence of pregnancy after the transfer of a total of 5 high-quality embryos is the inclusion criterion in the study. As regards uterine fibroids, they are not an exclusion criterion in the study.

## Conclusions

Metagenomics and traditional methods share some limitations, while also complement each other. Thus, this creates new diagnostic potential for research. The microbiota in the uterine cavity has been much less characterized. The upper female genital tract, consisting of the uterus, fallopian tubes and ovaries, was once considered a sterile environment. Although this approach has fundamentally changed over the years, there is still no current consensus on the basic female genital tract microbiota occurring in healthy women, nor its exact role in the formation of uterine fibroids. However, an increasing amount of strong evidence is available to support this changing concept.

This systematic review presents the relationship between intestinal and uterine dysbiosis and uterine fibroids. In recent years, researchers dealing with reproduction in a broad sense have focused on the microbiome in various locations to study its role in the pathogenesis and, consequently, the prevention and treatment of diseases of the genital organ. Recent studies of microbiomes of different locations have identified patterns of the bacterial composition of these sites depending on such factors as the population. The next step involves increasingly advanced research on health changes related to specific conditions, especially with regard to the impact of steroid hormone axes, and the interaction of estrogens with the gut microbiome.

Based on available data, it may be concluded that the gut and uterine microbiome are related and that the role of this microbiome is greater than expected. Numerous pathogenetic pathways are shared by the microbiome and fibroids, which allows a conclusion that the presence of fibroids or some symptoms may be dependent on changes in the microbiome. In view of the few results on the link between the microbiome and uterine fibroids, further intensive studies in humans and animal models are necessary, including the possible use of different microbiome modulations in the prevention or treatment of uterine fibroids.

## Author contributions

Conceptualization, MC, NZ-L; Methodology, MZ; Search for references, MZ, NZ-L, LK; Writing—original draft preparation, NZ-L, MC, LK; Writing—review and editing, NZ-L, LK, MZ, EZ, KZ, CW, MD; Visualization, NZ-L, MZ; Supervision, MC. All authors contributed to the article and approved the submitted version.
